# A Three-Dimensional Target Localization Method for Satellite–Ground Bistatic Radar Based on a Geometry–Motion Cooperative Constraint

**DOI:** 10.3390/s25113568

**Published:** 2025-06-05

**Authors:** Fangrui Zhang, Hu Xie, She Shang, Hongxing Dang, Dawei Song, Zepeng Yang

**Affiliations:** Xi’an Institute of Space Radio Technology, Xi’an 710100, China; zhang_fr0601@163.com (F.Z.);

**Keywords:** satellite–ground bistatic radar, geometry–motion cooperative constraint, variable step-size segmentation search, reduced-dimension projection

## Abstract

This paper investigates the three-dimensional target localization problem in satellite–ground bistatic radar. In conventional bistatic radar systems, passive receivers struggle to directly acquire the altitude information of the target, making it difficult to achieve effective three-dimensional target localization. This paper uses the bistatic distance data obtained after signal processing to construct ellipsoidal constraints, thereafter combining azimuth data to compress the position solution space into a three-dimensional elliptical line. Introducing the assumption of short-term linear uniform motion of the target, the target trajectory and elliptical line constraints are projected onto a two-dimensional plane, establishing an optimization model to determine the target trajectory parameters, ultimately yielding the target’s three-dimensional coordinates and completing the positioning process. The simulation results demonstrate the efficacy and performance of the proposed method.

## 1. Introduction

Radar target tracking and localization have extensive applications in both civilian and military domains. Space-based early warning radar is crucial for tracking and positioning because of its capability to detect diverse targets. It can effectively make up for the shortcomings of existing early warning radar systems. Space-based early warning radar is essentially a bistatic/multi-base radar system. It uses satellites as transmitters and sets up one or more receivers to reconnaissance specific areas. The distinctive architecture of separate transmitters and receivers, along with inherent advantages in concealment and multi-angle detection, has rendered the target positioning of space-based early warning radar systems a prominent focus of research. The positioning method often relies on the joint measurement of time difference of arrival (TDOA), frequency difference of arrival (FDOA), or phase difference by multiple stations and then developing linear or nonlinear models to determine the target position. Current research methods can be classified into two categories: direct and indirect. The target parameter information of the direct method is directly estimated from the received echo. In 2011, Bar-Shalom et al. introduced the direct position determination (DPD) method [1] into the MIMO (Multiple Input–Multiple Output) radar system and gave a maximum likelihood algorithm model for stationary target position estimation [2]. In [3], Zhang et al. studied the problem of noncoherent distributed MIMO radar positioning under bandwidth-limited conditions. Given the huge transmission load of the sampling signal in the traditional direct positioning algorithm, the baseband echo was low-bit quantized at each receiving end, and then the maximum likelihood estimator was used to obtain the target position.

The indirect approach is divided into two steps. The initial stage retrieves data from the echo. The second stage uses the measurement to solve the target position by formulating a linear or nonlinear equation. In 2007, Ho and Lu et al. proposed a two-stage target positioning method that simultaneously uses TDOA and FDOA measurement. Under the condition that the position is not accurately known, in addition to using FDOA measurement to obtain the additional ability to carry out target velocity estimation, it also improves the target positioning accuracy [4]. However, nonlinear interactions diminish the positioning accuracy dramatically as the platform position error rises. To address this problem, researchers employed first-order Taylor series expansion to establish a linear equation group. Then, they used the best linear unbiased estimator to obtain the target position, avoiding the position estimation error introduced by nonlinear terms [5,6]. In [7], assuming that the time delay between the transmitting and receiving antennas in the MIMO radar has been obtained, the authors established a linear equation between the received data and the unknown target position. They used the least squares method to estimate the target position. The simulation results show that the process has good positioning performance without relying on initial values. In [8], aiming to prevent the abnormal distance measurements that may occur in distributed MIMO radar systems, researchers used the maximum correlation entropy criterion to construct a cost function and then used a semi-quadratic optimization method to solve the nonconvex problem. Simulation experiments proved the robustness of this method.

Furthermore, certain researchers have employed distance measurements to formulate a nonconvex weighted least squares problem for the simultaneous estimation of transmitter and target positions, utilizing semidefinite relaxation (SDR) to transform the problem into a convex semidefinite programming framework [9]. Certain scholars have also presented a Lagrangian programming neural network method for addressing nonlinear constrained optimization problems, grounded in Lagrangian multiplier theory [10,11].

While the direct method can intuitively acquire the required parameters, it poses significant challenges in terms of system computational load. Currently, indirect methods are predominantly employed to address the distributed radar target positioning issue. Researchers have devised many constrained optimization techniques. Nevertheless, the majority of these optimization techniques necessitate precise initial values in the first phase to guarantee estimation accuracy. Research on 3D target localization for dispersed multi-base radar systems has reached a considerable level of maturity; nonetheless, certain challenges remain that are difficult to address. First, the stringent temporal synchronization across numerous station elevates the complexity [12,13,14]; second, three-dimensional positioning requires that the spatial layout of multiple stations meet certain geometric observable conditions, that is, reasonable resource allocation and scheduling are required for different application scenarios, otherwise it may cause positioning ambiguity or ill-conditioned solution problems [15,16,17,18]; third, in real detection circumstances, the localization of airborne objects is influenced by the number of receiving stations, and the actual observation region is frequently monitored by only a single station [19]. Currently, the multi-base radar system degenerates into a bistatic configuration including one transmitter and one receiver. Inadequate model input information may impair the efficacy of the above positioning methods or may even result in incorrect resolution of the 3D coordinates.

Based on this premise, researchers have explored 3D target localization within the framework of bistatic radar. Nevertheless, the challenge of passive receivers directly obtaining target altitude [20] has resulted in inadequate information for bistatic radar positioning. To address the issue, certain researchers have incorporated additional information by modifying the layout of the transmitting/receiving array to assist positioning. In [21], Cheng et al. employed a uniform rectangular planar array arrangement and utilized tensor decomposition to derive the parameter matrix. They calculated the Doppler frequency, direction of departure (DoD), and direction of arrival (DoA), utilizing the properties of the Vandermonde structure. In [22], Li et al. employed a configuration comprising a transmitting uniform circular array and a receiving uniform linear array to ascertain the target’s azimuth, elevation angle, and receiving cone angle from the received data. Without bistatic radar distance data, Li utilized angular information to ascertain the target’s 3D coordinates. Several works have written on the topic of the localization of bistatic MIMO radar [23,24,25,26,27].

To enhance the practicality of bistatic radar positioning, this paper aims to conduct a more in-depth analysis of the measurement data instead of modifying the array layout. In [20], the author presented a passive bistatic radar target localization method utilizing multiple receivers and non-cooperative multiple transmitters. The approach utilizes the bistatic distances of various transmitters and receivers to construct an ellipsoid, thereafter finding the intersecting points of these ellipsoids in three-dimensional space to locate the position of the target. In [28], the author added angle-of-arrival (AOA) data based on bistatic distance and performed target 3D localization through two-stage processing. Similar work was carried out in [29,30,31]. The distinguishing feature of [20,28,29,30,31] is their reliance measurement data as input for data-level target localization, but mostly based on multi-base configurations.

Building upon the aforementioned research, this paper introduces a 3D target localization method under bistatic radar by further utilizing measurement information and motion characteristics. This paper uses measured bistatic distance data to establish an ellipsoidal constraint, subsequently compressing the solution space into a three-dimensional ellipse by combining the azimuthal tangent plane. The hypothesis of the short-term uniform linear motion of the target is introduced. Subsequently, a three-dimensional ellipse and the target’s motion trajectory are projected onto a two-dimensional plane. The slope of the trajectory is analytically determined by constructing a cost function utilizing the relative time difference of motion, while the intercept range is constrained. A variable step-size segmentation search method is designed, incorporating the curvature criterion, to accurately search intercept solution and three-dimensional coordinates, ultimately achieving three-coordinate positioning in a single transmitter and single receiver configuration.

The main innovations of the paper are as follows:

(1) The method of projecting the three-dimensional ellipse and the target motion trajectory onto a two-dimensional plane, while determining the trajectory slope through the construction of a cost function, circumvents the substantial computational complexity associated with three-dimensional solution searches. The framework of “reducing dimension–solving–increasing dimension” is quite uncommon in the positioning algorithms found in the current literature.
(2) The integration of the intercept search mechanism of variable step-size segmentation and curvature criterion guarantees computational efficiency and accuracy, rendering it more applicable in engineering than iterative optimization methods.

The rest of the paper is organized as follows. Section 2 discusses in detail the target localization method using bistatic distance and azimuth measurement; to verify the effectiveness of the proposed method, Section 3 gives simulation experiments and analyzes the ranging accuracy with different measurement errors; finally, Section 4 summarizes the paper and offers conclusions.

## 2. Positioning Model

**Figure 1:**
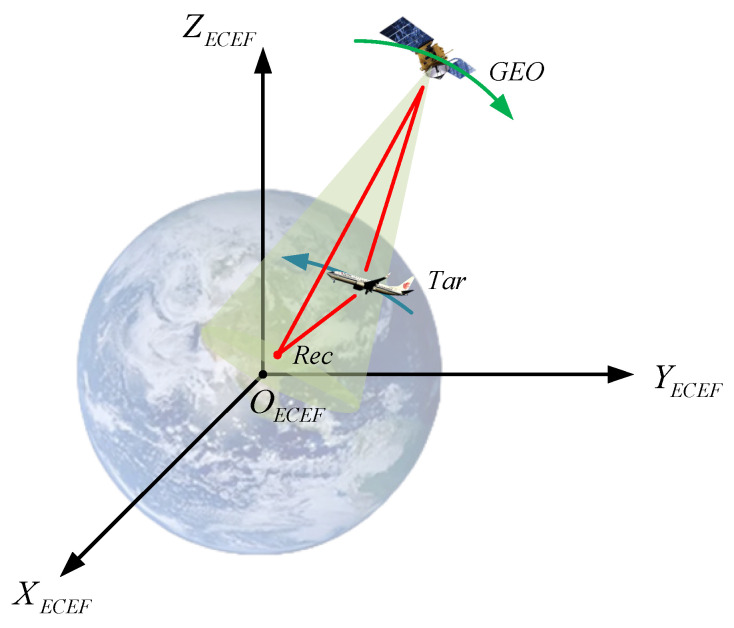
Geometric configuration of satellite–ground bistatic radar system.

Consider a bistatic radar system with a GEO satellite as the transmitter and a uniform linear array as the receiver, as shown in Figure 1. The coordinates of the GEO satellite and the ground receiver are known in the earth-centered earth-fixed (ECEF) coordinate system, which are $geo\left(X_{geo},Y_{geo},Z_{geo}\right)$ and $rec\left(X_{rec},Y_{rec},Z_{rec}\right)$. The coordinate of the target, which is $tar\left(X_{tar},Y_{tar},Z_{tar}\right)$, is unknown.

### 2.1. Position Ellipsoid Constraint

Assume that the receiver has obtained the measurement of the bistatic distance ΔR and the azimuth $\theta_{tar}$ of the target relative to the receiver after signal processing. The bistatic distance ΔR is

(1) $$\Delta R=R_{geo\_tar}+R_{rec\_tar}$$

In the above formula, $R_{geo\_tar}$ is the distance between the transmitter and the target, and $R_{rec\_tar}$ is the distance between the receiver and the target. The position of the target at any time is on an ellipsoid with the transmitter and the receiver as the focus and the bistatic distance ΔR as the major axis length. Considering the difficulty of directly solving the explicit equation of the target position ellipsoid, a virtual standard ellipsoid equation is first established at the ECEF coordinate origin, and then the ellipsoid is transformed to its actual position using coordinate transformation. The standard ellipsoid equation in the ECEF coordinate system is given as follows:

(2) $$\frac{x^{2}}{a^{2}}+\frac{y^{2}}{b^{2}}+\frac{z^{2}}{c^{2}}=1$$

In the above formula,

(3) $$\left\{\begin{array}{l} a=\frac{\Delta R}{2}\\ b=c=\sqrt{a^{2}-{\left(\frac{R_{geo\_rec}}{2}\right)}^{2}} \end{array}\right.$$

In the above formula, $R_{geo\_rec}$ is the distance between transmitter and receiver. The coordinate transformation of the above standard ellipsoid includes two parts: rotation and translation. The rotation matrix is recorded as

(4) $$T=\left[\begin{array}{ccc} T_{1} & T_{2} & T_{3} \end{array}\right]$$

In the above formula, $T_{1}$ is the normalized direction vector of the line connecting the transmitter and the receiver, and the direction is from the transmitter to the receiver.

(5) $$T_{1}={\left[\frac{geo\left(1\right)-rec\left(1\right)}{{\left\|geo-rec\right\|}_{2}},\frac{geo\left(2\right)-rec\left(2\right)}{{\left\|geo-rec\right\|}_{2}},\frac{geo\left(3\right)-rec\left(3\right)}{{\left\|geo-rec\right\|}_{2}}\right]}^{\prime }$$

In the above formula, ${\left\|\cdot \right\|}_{2}$ representing the 2-norm, $T_{2}$ and $T_{3}$ are a set of unit vectors that are orthogonal to $T_{1}$. The solution steps are summarized in Table 1, as shown below.

The translation vector is

(6) $$t={\left[\frac{geo+rec}{2}\right]}^{\prime }$$

The ellipsoid transformation relationship is

(7) $$\left[\begin{array}{c} x^{\prime }\\ y^{\prime }\\ z^{\prime } \end{array}\right]=T\left[\begin{array}{c} x\\ y\\ z \end{array}\right]+t$$

In the above formula, $\left(x,y,z\right)$ and $\left(x^{\prime },y^{\prime },z^{\prime }\right)$ represent the points on the ellipsoid before and after the transformation, respectively. The equation of the ellipsoid with the transmitter and the receiver as the double focus is

(8) $$\begin{array}{l} \frac{{\left(T_{11}\left(x^{\prime }-t_{x}\right)+T_{12}\left(y^{\prime }-t_{y}\right)+T_{13}\left(z^{\prime }-t_{z}\right)\right)}^{2}}{a^{2}}+\\ \frac{{\left(T_{21}\left(x^{\prime }-t_{x}\right)+T_{22}\left(y^{\prime }-t_{y}\right)+T_{23}\left(z^{\prime }-t_{z}\right)\right)}^{2}}{b^{2}}+\\ \frac{{\left(T_{31}\left(x^{\prime }-t_{x}\right)+T_{32}\left(y^{\prime }-t_{y}\right)+T_{33}\left(z^{\prime }-t_{z}\right)\right)}^{2}}{b^{2}}=1 \end{array}$$

### 2.2. Position Ellipse Constraint

All points with an azimuth angle of $\theta_{tar}$ relative to the receiver $rec\left(X_{rec},Y_{rec},Z_{rec}\right)$ form an azimuthal tangent plane, which is recorded as

(9) $$\gamma =\left\{\left.\left(rec\left(1\right)+N\cdot \cos\left(\theta_{tar}\right),\;rec\left(2\right)+N\cdot \sin\left(\theta_{tar}\right),\;Z\right)\right|N,Z\in \mathbb{R}\right\}$$

In Formula (9), if *N* and *Z* are any real numbers, the azimuth angle of the obtained point relative to $rec\left(X_{rec},Y_{rec},Z_{rec}\right)$ is $\theta_{tar}$. By taking all *N* and *Z* values, the set of all points forms the plane γ. Take any two points on the tangent plane and record their coordinates as follows:

(10) $$\left\{\begin{array}{l} P_{1}\left(rec\left(1\right)+N_{1}\cos\left(\theta_{tar}\right),\;rec\left(2\right)+N_{1}\sin\left(\theta_{tar}\right),\;Z_{1}\right)\\ P_{2}\left(rec\left(1\right)+N_{2}\cos\left(\theta_{tar}\right),\;rec\left(2\right)+N_{2}\sin\left(\theta_{tar}\right),\;Z_{2}\right) \end{array}\right.$$

Combine the receiver coordinates $rec\left(X_{rec},Y_{rec},Z_{rec}\right)$ to obtain the azimuthal tangent plane equation:

(11) $$\left|\begin{array}{ccc} x-rec\left(1\right) & y-rec\left(2\right) & z-rec\left(3\right)\\ P_{1}\left(1\right)-rec\left(1\right) & P_{1}\left(2\right)-rec\left(2\right) & P_{1}\left(3\right)-rec\left(3\right)\\ P_{2}\left(1\right)-rec\left(1\right) & P_{2}\left(2\right)-rec\left(2\right) & P_{2}\left(3\right)-rec\left(3\right) \end{array}\right|=0$$

**Figure 2:**
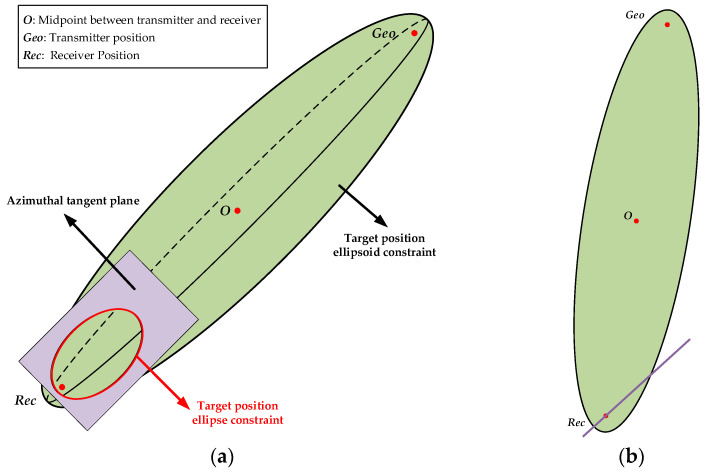
Schematic diagram of the intersecting line of the ellipsoid and the azimuthal tangent plane: (**a**) side view and (**b**) top view.

By combining Equations (8) and (11), we can obtain the ellipse intersecting line equation $f\left(x,y,z\right)$ in each azimuth direction. The intersecting relationship between the ellipsoid constraint and the azimuthal tangent plane constraint is shown in Figure 2.

### 2.3. Assumption of Short-Term Linear Motion of the Target

High-speed targets in cruise mode can be regarded as an ideal uniform linear motion model in a very short period of time. The spatial line equation of the target motion trajectory is assumed to be

(12) $$\left\{\begin{array}{l} x\left(t\right)=x_{0}+v_{x}t\\ y\left(t\right)=y_{0}+v_{y}t\\ z\left(t\right)=z_{0}+v_{z}t \end{array}\right.$$

where t is understood as time, and the direction vector $\left(v_{x},v_{y},v_{z}\right)$ is understood as speed. Assume that the target reaches $P_{1}\left(x_{0}+v_{x}t_{1},y_{0}+v_{y}t_{1},z_{0}+v_{z}t_{1}\right)$ at time $t_{1}$, $P_{2}\left(x_{0}+v_{x}t_{2},y_{0}+v_{y}t_{2},z_{0}+v_{z}t_{2}\right)$ at time $t_{2}$, and $P_{3}\left(x_{0}+v_{x}t_{3},y_{0}+v_{y}t_{3},z_{0}+v_{z}t_{3}\right)$ at time $t_{3}$. The ratio of distances satisfies the following relationship:

(13) $$\frac{{\left\|P_{1}P_{3}\right\|}_{2}}{{\left\|P_{1}P_{2}\right\|}_{2}}=\frac{\sqrt{v_{x}^{2}{\left(t_{3}-t_{1}\right)}^{2}+v_{y}^{2}{\left(t_{3}-t_{1}\right)}^{2}+v_{z}^{2}{\left(t_{3}-t_{1}\right)}^{2}}}{\sqrt{v_{x}^{2}{\left(t_{2}-t_{1}\right)}^{2}+v_{y}^{2}{\left(t_{2}-t_{1}\right)}^{2}+v_{z}^{2}{\left(t_{2}-t_{1}\right)}^{2}}}=\frac{t_{3}-t_{1}}{t_{2}-t_{1}}$$

**Figure 3:**
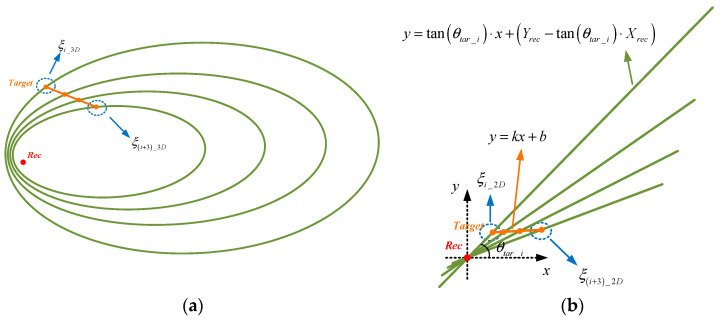
Intersecting line diagram: (**a**) ellipse constraint and target trajectory and (**b**) 2D projection.

Under uniform motion conditions, the ratio of the distances among any three points is only proportional to the relative time difference of the target’s movement. The ellipse formed by the intersecting line of the azimuthal tangent plane and the target position ellipsoid, and the target motion trajectory are both projected onto a plane perpendicular to the azimuthal tangent plane, resulting in a two-dimensional intersecting line, as shown in Figure 3.

As shown in Figure 3b, the equation for the target trajectory in the projection plane is presumed to be

(14) y=kx+b

**Figure 4:**
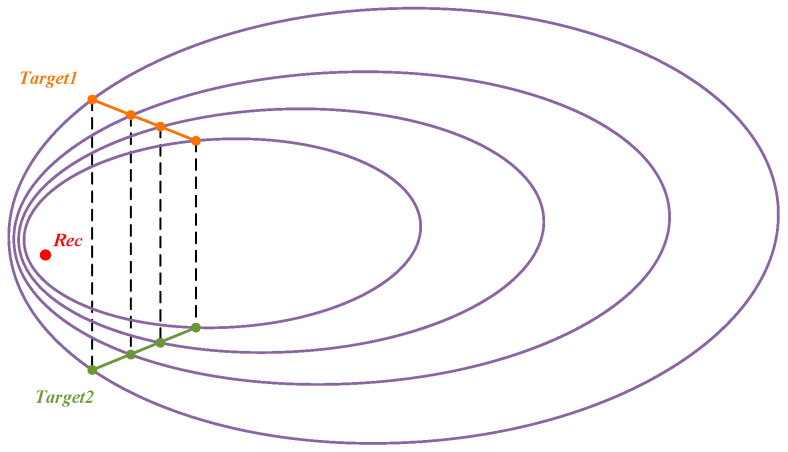
Illustration of feasible solutions that satisfy the straight line trajectory relationship.

Should the parameters ***k*** and ***b*** in Equation (14) be determinable, then two sets of positions will fulfill the linear relationship depicted in Figure 3b, as illustrated in Figure 4. The erroneous solution can be discarded based on the elevation angle relationship between the two positional sets and the receiver. The positioning problem is transformed into determining the unknown parameters ***k*** and ***b***.

#### 2.3.1. Solution for the Slope k of Trajectory

The projection equation of the ellipse intersecting line is

(15) $$y=\tan\left(\theta_{tar\_i}\right)\cdot x+\left(Y_{rec}-\tan\left(\theta_{tar\_i}\right)\cdot X_{rec}\right)$$

In Formula (15), the subscript “*i*” is used to distinguish the ellipse intersecting lines at different azimuths. Then, the two-dimensional coordinate $\xi_{i\_2D}$ of the target at azimuth $\theta_{tar\_i}$ is

(16) $$\xi_{i\_2D}\left(\frac{b-\left(Y_{rec}-\tan\left(\theta_{tar\_i}\right)X_{rec}\right)}{\tan\left(\theta_{tar\_i}\right)-k},\frac{b-\left(Y_{rec}-\tan\left(\theta_{tar\_i}\right)X_{rec}\right)}{\tan\left(\theta_{tar\_i}\right)-k}k+b\right)$$

In Formula (16), only ***k*** and ***b*** are unknown parameters. The cost function is formulated in conjunction with Formula (13) using the five adjacent target positions:

(17) $$\begin{array}{ll} f\left(k,b\right) & ={\left(\frac{{\left\|\xi_{i\_2D}\xi_{\left(i+2\right)\_2D}\right\|}_{2}}{{\left\|\xi_{i\_2D}\xi_{\left(i+1\right)\_2D}\right\|}_{2}}-\frac{t_{\left(i+2\right)}-t_{i}}{t_{\left(i+1\right)}-t_{i}}\right)}^{2}+{\left(\frac{{\left\|\xi_{i\_2D}\xi_{\left(i+3\right)\_2D}\right\|}_{2}}{{\left\|\xi_{i\_2D}\xi_{\left(i+1\right)\_2D}\right\|}_{2}}-\frac{t_{\left(i+3\right)}-t_{i}}{t_{\left(i+1\right)}-t_{i}}\right)}^{2}\\ & +{\left(\frac{{\left\|\xi_{i\_2D}\xi_{\left(i+4\right)\_2D}\right\|}_{2}}{{\left\|\xi_{i\_2D}\xi_{\left(i+1\right)\_2D}\right\|}_{2}}-\frac{t_{\left(i+4\right)}-t_{i}}{t_{\left(i+1\right)}-t_{i}}\right)}^{2} \end{array}$$

By minimizing the cost function $\underset{k}{\min}\;f\left(k,b\right)$, the slope ***k*** of the target trajectory can be obtained.

#### 2.3.2. Solution of the Intercept b of Trajectory

There is only one optimal intercept ***b*** in three-dimensional space, which aligns the neighboring target points into a straight line to the greatest extent feasible. The paper takes the total curvature as the cost function and proposes a variable step-size segmentation method to determine the optimal ***b***. Total curvature is defined as the aggregate of the angular changes between adjacent points. Taking the four points $\xi_{1}$, $\xi_{2}$, $\xi_{3}$, and $\xi_{4}$ as an example, the total curvature Θ is defined as

(18) $$\Theta =\arccos\left(\frac{\xi_{1}\xi_{2}\cdot \xi_{2}\xi_{3}}{{\left\|\xi_{1}\xi_{2}\right\|}_{2}\cdot {\left\|\xi_{2}\xi_{3}\right\|}_{2}}\right)+\arccos\left(\frac{\xi_{2}\xi_{3}\cdot \xi_{3}\xi_{4}}{{\left\|\xi_{2}\xi_{3}\right\|}_{2}\cdot {\left\|\xi_{3}\xi_{4}\right\|}_{2}}\right)$$

The solution process of variable step-size segmentation search is shown in Table 2.

**Figure 5:**
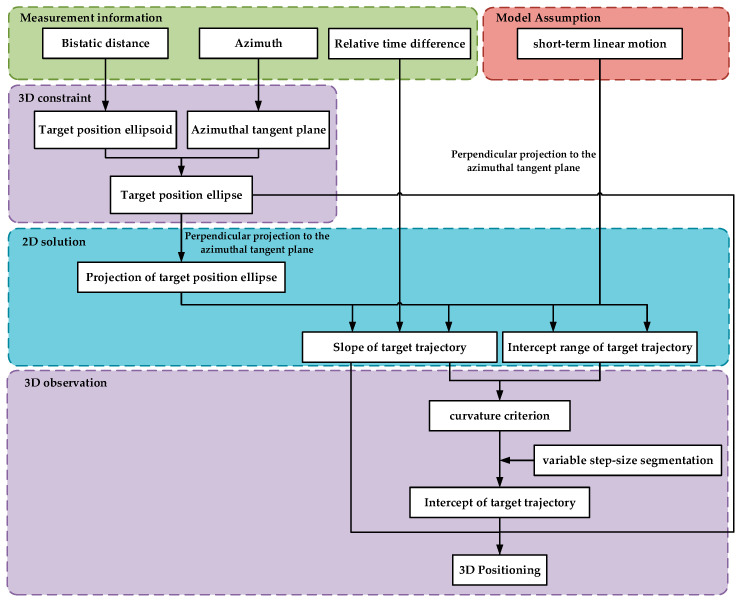
Overall target positioning framework.

The overall positioning framework is shown in Figure 5.

## 3. Results

**Figure 6:**
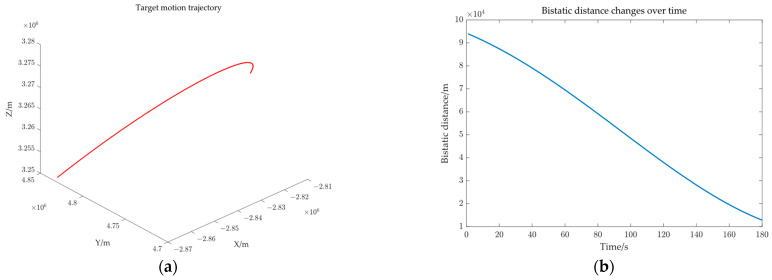
Target to be detected: (**a**) target motion trajectory and (**b**) the curve of the bistatic distance changing over time.

Figure 5 illustrates that the input data for three-dimensional target positioning comprises three components: the bistatic distance, the target’s azimuth relative to the receiver, and the relative time difference of the target motion. The paper assumed that the relative time difference can be accurately measured, primarily examining the influence of measurement errors introduced by the bistatic distance and the azimuth on positioning accuracy. Section 3 considers three scenarios: only the bistatic distance measurement error exists, only the azimuth angle measurement error exists, and both the bistatic distance and azimuth angle measurement errors exist simultaneously. Under identical conditions, the unscented Kalman filter (UKF) [32] and the particle Filter (PF) [33] are introduced as comparison methods. The radar transmitter is installed on a GEO satellite, while the receiver is placed on the ground to detect civil aircraft via passive reception. Assume that the azimuth variation of the detected target relative to the receiver over 180 s is $\left(10,\;80\right)$ degrees, the elevation variation is $\left(33,\;62\right)$ degrees, and the positional variation is $\left(116,\;149\right)$ kilometers, as shown in Figure 6. The simulation experiment parameters are set as shown in Table 3.

The paper uses the root mean square error (RMSE) as an indicator to evaluate the accuracy of the positioning method, focusing on both range and elevation measurement. The formula for calculating RMSE is as follows:

(19) $$RMSE=\sqrt{\frac{1}{N}\sum_{i=1}^{N}{\left({\hat{r}}_{i}-r_{i}\right)}^{2}}$$

In the above formula, N represents the number of samples, ${\hat{r}}_{i}$ represents the estimated value, and $r_{i}$ represents the theoretical value.

### 3.1. Explanation on the Assumption of Short-Term Linear Motion

**Figure 7:**
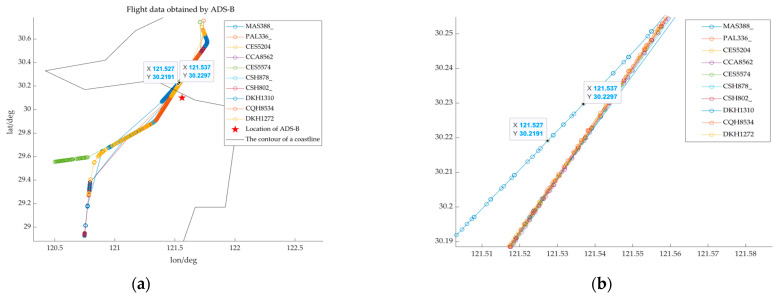
Flight data obtained by ADS-B. (**a**) Flight data obtained by ADS-B. (**b**) Local zoom.

In this study, we conducted detection experiments utilizing ADS-B (Automatic Dependent Surveillance-Broadcast) equipment at civil airports to validate the assumption of “short-term linear motion”. Figure 7 illustrates the experimental outcomes.

The cruising speed of the commercial aircraft is about 260 m/s, resulting in a trajectory length of 1040–1300 m over a duration of 4–5 s. Figure 7 illustrates the flight trajectories of various aircraft, with the horizontal axis denoting longitude and the vertical axis indicating latitude. We identified the coordinates (121.527, 30.2191) and (121.537, 30.2297) and compute the distance between them, which is 1520.64 m. The path between the two points in Figure 7b represents the aircraft’s movement over a brief interval of 4–5 s. According to Figure 7 and the calculation results, it can be inferred that the civil aircraft exhibits uniform linear motion for a brief duration of 4 to 5 s.

### 3.2. Analysis of the Influence of Bistatic Distance Error

When there is a measurement error Δr of the bistatic distance, Equation (3) can be rewritten as

(20) $$\left\{\begin{array}{l} a=\frac{\Delta R+\Delta r}{2}\\ b=c=\sqrt{a^{2}-{\left(\frac{R_{geo\_rec}}{2}\right)}^{2}} \end{array}\right.$$

**Figure 8:**
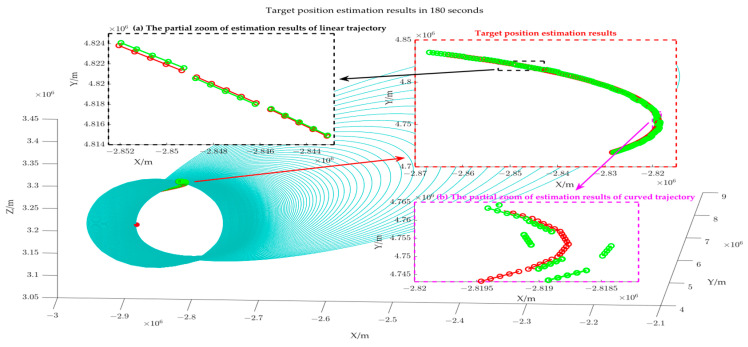
Target position estimation results when the bistatic distance error is 200 m.

From a geometric perspective, the error Δr will alter the lengths of the major and minor axes of the target position’s ellipsoid, but the two foci of the ellipsoid remain invariant, equivalently resulting in an overall scaling of the ellipsoid. In the absence of azimuthal error, the slope of the projection line of the ellipse constraint is accurate, and the slope ***k*** of the projection line of the target trajectory is accurate; however, the scaling of the ellipsoid results in a deviation of the intercept ***b*** of the target trajectory from the optimal solution, thereby affecting the calculation accuracy of the target points. It is worth noting that the length of the major and minor axes (about $10^{7}$) is significantly larger than the bistatic distance measurement error, allowing for a certain tolerance for bistatic distance measurement error. Figure 8 shows the target position estimation results at 180 points.

Figure 8 illustrates that the blue loops represent the elliptical line constraint formed by the intersection of several azimuthal tangent planes and the different ellipsoid constraint, while the green trajectory is the estimation result of our paper, and the red trajectory is the theoretical target position. It can be observed that given the assumption of short-term linear target motion, the position estimation error at the target’s turning point is significant, as illustrated in Figure 8b; when the target motion is relatively gentle, the estimation deviation is minimal, as shown in Figure 8a.

**Figure 9:**
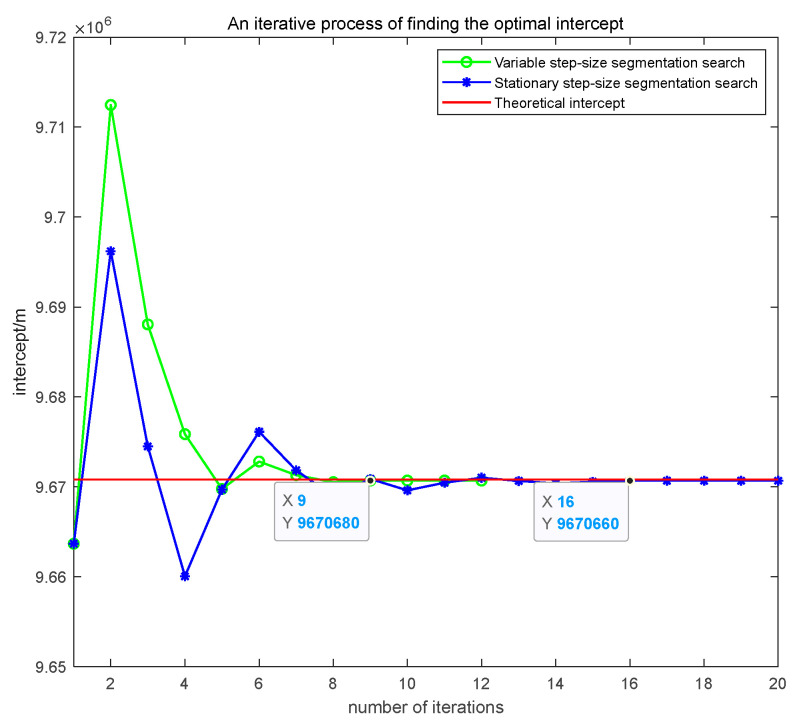
The iterative process of finding the optimal intercept.

As shown in Figure 9, this paper presents the comparative results of the convergence curves between the variable step-size segmentation optimization method proposed in this paper and the fixed step-size optimization method under the conditions of bistatic distance; the measured error is 250 m. The green line represents the variable step-size segmentation optimization method, the blue line represents the fixed step-size optimization method, and the red line represents the theoretical intercept of a short-term linear trajectory. The method proposed in this paper achieved convergence to the optimal solution by the ninth iteration, with an error of 115.3750 m and a duration of 7.38 s.

Under identical error conditions, the fixed step-size method converged to the optimal solution in the 16th iteration, with an error of 135.1320 m and a duration of 17.71 s. The method proposed in the paper exhibits faster convergence speed and better convergence accuracy. This is because in the initial stage, a large step length is used to determine the optimal solution interval, and in the later stage, a small step length is used for precise optimization, which accelerates the convergence speed while ensuring convergence.

**Figure 10:**
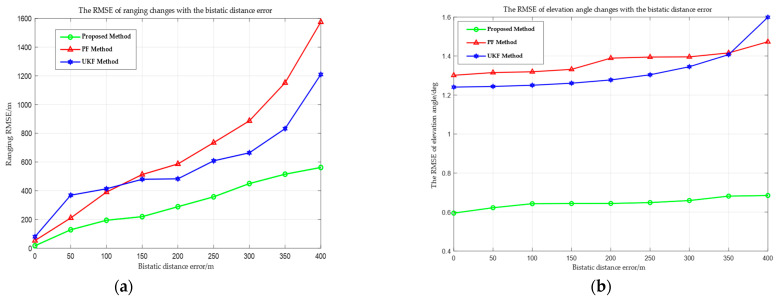
RMSE changes with the bistatic distance errors. (**a**) RMSE of ranging. (**b**) RMSE of elevation angle measurement.

Figure 10 shows the RMSE curves for range and elevation angle measurement of the proposed method, UKF, and PF across various bistatic distance errors. With the increase in the bistatic distance error, the RMSE of all three methods rises; nevertheless, the error growth trend of the proposed method is markedly less than UKF and PF. For instance, when the bistatic distance error is 300 m, the RMSE of the proposed method is 449.83 m, whereas the UKF reaches 664.12 m and the PF reaches 886.68 m. This is because the two observational parameters (the bistatic distance and the azimuth angle) cannot uniquely determine the three degrees of freedom of position, particularly in the Z direction. The height information cannot be directly observed, resulting in obvious position estimation errors. In contrast, the proposed method uses geometric structure and short-term linear motion to establish a “strong constraint”, effectively mitigating measurement data errors and preventing long-term state estimation from drifting due to error accumulation. However, it is evident from the comparison of Figure 10a,b that the RMSE growth trend for ranging grows significantly more than that for elevation angle measurement when the bistatic distance error increases. The strong coupling relationship between the three-dimensional coordinates of the target position and the bistatic distance error Δr is the reason for this. The error Δr will directly impact the solution of the three-dimensional coordinates via the ellipsoid equation constraint, hence exacerbating the ranging error. At the same time, the elevation angle is only associated with the Z coordinate solution and is indirectly affected by Δr, resulting in a little impact.

### 3.3. Analysis of the Influence of Azimuth Error

When there is an azimuth angle measurement error Δθ, Equation (11) can be rewritten as

(21) $$\left|\begin{array}{ccc} x-rec\left(1\right) & y-rec\left(2\right) & z-rec\left(3\right)\\ N_{1}\cos\left(\theta_{tar}+\Delta \theta \right) & N_{1}\sin\left(\theta_{tar}+\Delta \theta \right) & P_{1}\left(3\right)-rec\left(3\right)\\ N_{2}\cos\left(\theta_{tar}+\Delta \theta \right) & N_{2}\sin\left(\theta_{tar}+\Delta \theta \right) & P_{2}\left(3\right)-rec\left(3\right) \end{array}\right|=0$$

**Figure 11:**
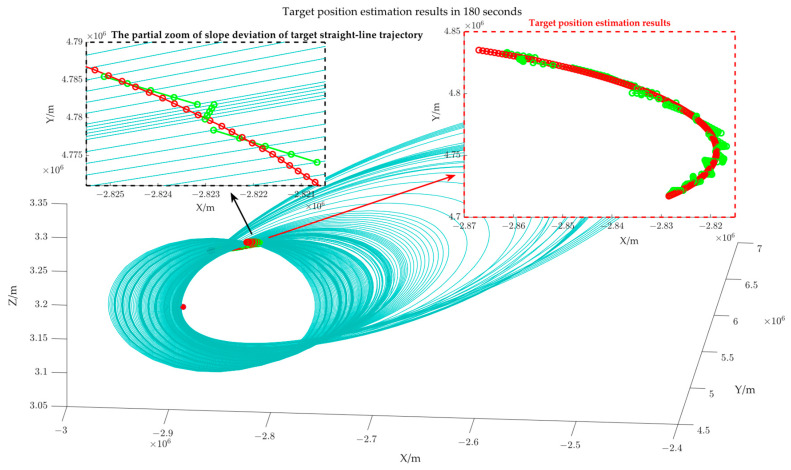
Target position estimation results when the azimuth angle measurement error is 2 degrees.

**Figure 12:**
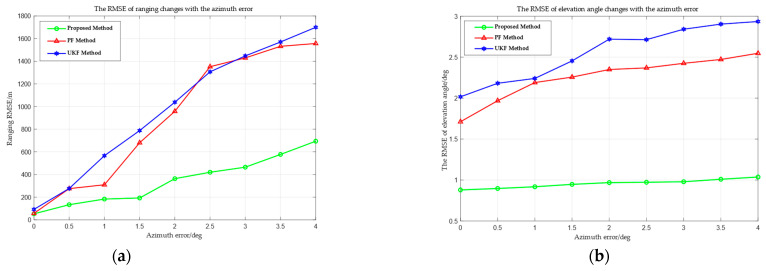
RMSE changes with the azimuth angle measurement errors. (**a**) RMSE of ranging. (**b**) RMSE of elevation angle measurement.

Figure 11 shows the target position estimation results when the azimuth measurement error is 2 degrees. The blue loops represent the elliptical line constraint formed by the intersection of several azimuthal tangent planes and different ellipsoid constraints; the green trajectory is the estimated result of the study; and the red trajectory is the theoretical target position. Upon comparing Figure 8 and Figure 11, it is evident that the error Δθ leads to an irregular distribution of the elliptical line constraint, thereby affecting the solution of the target trajectory projection slope (as shown in Figure 11), which further exacerbates target position error. The change in the RMSE in relation to varied azimuth angle measurement errors is shown in Figure 12.

Figure 12 shows the RMSE curves of ranging and elevation angle measurements of the proposed method, UKF, and PF, with varying azimuth angle measurement errors. The amalgamation of Figure 10 and Figure 12 reveals that the azimuth angle measurement errors lead to deviations in the elliptical line constraint. This further leads to inaccuracies in the projected slope of the target short-term line trajectory (as shown in Figure 11). Meanwhile, to meet the strong constraint of “short-term linear motion”, the optimal intercept determined under the incorrect slope and the ultimately computed three-dimensional coordinates of the target position also exhibit inaccuracies.

### 3.4. Simultaneous Analysis of the Influence of Bistatic Distance Error and Azimuth Error

To evaluate the influence of the azimuth angle error and the bistatic distance error on positioning accuracy, this paper used an analysis of variance (ANOVA) to quantify the contribution of different measurement errors to the RMSE of ranging. An experiment utilizing control variables is structured to examine the independent contribution and interactions of two measurement errors on the overall error in target positioning. The two measurement errors are categorized into three levels—low, medium, and high—as shown in Table 4 and Table 5.

The experimental parameters used in Section 3.4, encompassing the coordinates of the transmitter and the receiver, and the actual trajectory of the target align with those presented in the preceding two sections. Considering nine experimental combinations under different error conditions, each group of experiments involved 50 Monte Carlo simulations, and the RMSE of ranging of each simulation was recorded as the dependent variable. Table 6 presents the mean ranging RMSE for all error conditions.

**Figure 13:**
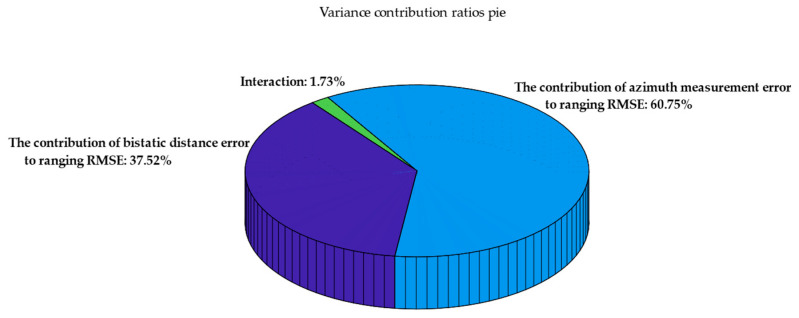
Pie chart of variance contribution ratios.

All the above data were analyzed to generate a pie chart of variance contribution ratios, as shown in Figure 13.

Figure 13 shows the contribution of each component of the measurement error and their interactions to the variation of the ranging RMSE. The azimuth angle measurement error constitutes the largest share at 60.75%, signifying that the ranging RMSE is most susceptible to it; the bistatic distance error contributes 37.52%, and there exists a synergistic effect between the bistatic distance error and the azimuth angle measurement error, suggesting that the simultaneous occurrence of both errors may further compromise ranging accuracy. The interaction accounts for 1.73%. Systematically assessing the impact of each mistake source can effectively guide subsequent algorithm design and optimization.

## 4. Conclusions

The paper proposes a three-dimensional target positioning method for satellite–ground bistatic radar. The integration of geometric constraints and motion models effectively addresses the reliance of traditional positioning systems on multiple receivers. The bistatic distance constructs the target position ellipsoidal constraint, and the target position solution space is compressed to a three-dimensional ellipse line by combining the azimuthal tangent plane; then, a short-term linear motion hypothesis is further introduced, and the slope of the target trajectory is solved through dimensionality reduction projection. The variable step-size segmentation search method is further designed, combined with the curvature optimization function, to search the accurate intercept of the target trajectory and obtain the target’s three-dimensional position coordinates. In contrast to the multi-receiver positioning system, the proposed method provides an alternative feasible technical approach for resource-limited bistatic radar and holds significant application potential in aerospace surveillance. Future work will explore multi-target positioning capability in complex environments and optimize computational efficiency.

## Figures and Tables

**Table 1:** Rotation matrix solution steps.

Step 1: Let $T_{2}={\left[\begin{array}{ccc} T_{21} & T_{22} & T_{23} \end{array}\right]}^{\prime }$, where $T_{21}=1$, $T_{22}=0$. By $T_{2}\cdot T_{1}=0$, we get the expression for $T_{23}$:
$T_{23}=-\frac{geo\left(1\right)-rec\left(1\right)}{geo\left(3\right)-rec\left(3\right)}$
Step 2: $T_{3}$ is orthogonal to $T_{1}$ and $T_{2}$, and we can get the expression for $T_{3}$:
$T_{3}={\left[\frac{\left(geo\left(2\right)-rec\left(2\right)\right)\cdot \left(rec\left(1\right)-geo\left(1\right)\right)}{{\left\|geo-rec\right\|}_{2}\cdot \left(geo\left(3\right)-rec\left(3\right)\right)},\frac{geo\left(3\right)-rec\left(3\right)}{{\left\|geo-rec\right\|}_{2}},-\frac{geo\left(2\right)-rec\left(2\right)}{{\left\|geo-rec\right\|}_{2}}\right]}^{\prime }$
Step 3: Normalize $T_{2}$ and $T_{3}$.

**Table 2:** Variable step-size segmentation search solution process.

**Input:** 1. Intercept interval of the two-dimensional projection of the target’s short-term linear trajectory $\left[b_{left},b_{right}\right]$, slope ***k***; 2. Minimum threshold for intercept interval length ***th***; 3. Slope and intercept of the projection of the intersecting line of the ellipse at multiple adjacent points: $\tan\left(\theta_{tar\_i}\right)$, $\left(Y_{rec}-\tan\left(\theta_{tar\_i}\right)\cdot X_{rec}\right)$.
**Step 1:** Defining variable step size $h=\left(b_{right}-b_{left}\right)\cdot 10^{-6}$, find the total curvature of the line connecting of target positions at $b_{mid}=\left(b_{right}-b_{left}\right)/2$ and $\left(b_{mid}+h\right)$. Taking $b_{mid}$ as an example, the two-dimensional coordinate $\xi_{i\_2D\_mid}$ of the i-th target position can be found from Formula (16) as follows:
$\xi_{i\_2D\_mid}\left(\frac{b_{mid}-\left(Y_{rec}-\tan\left(\theta_{tar\_i}\right)X_{rec}\right)}{\tan\left(\theta_{tar\_i}\right)-k},\frac{b_{mid}-\left(Y_{rec}-\tan\left(\theta_{tar\_i}\right)X_{rec}\right)}{\tan\left(\theta_{tar\_i}\right)-k}k+b_{mid}\right)$
By solving the ellipsoid Equation (8), we can obtain the three-dimensional coordinates $\xi_{i\_3D\_mid}$ of the i-th target position. Similarly, we can obtain the three-dimensional coordinates $\xi_{\left(i+1\right)\_3D\_mid}$, $\xi_{\left(i+2\right)\_3D\_mid}$, and $\xi_{\left(i+3\right)\_3D\_mid}$ of the adjacent positions, and the corresponding total curvature is
$\begin{array}{ll} \Theta_{mid} & =\frac{\left(\xi_{\left(i+1\right)\_3D\_mid}-\xi_{i\_3D\_mid}\right)\cdot \left(\xi_{\left(i+2\right)\_3D\_mid}-\xi_{\left(i+1\right)\_3D\_mid}\right)}{{\left\|\xi_{\left(i+1\right)\_3D\_mid}-\xi_{i\_3D\_mid}\right\|}_{2}{\left\|\xi_{\left(i+2\right)\_3D\_mid}-\xi_{\left(i+1\right)\_3D\_mid}\right\|}_{2}}\\ & +\frac{\left(\xi_{\left(i+2\right)\_3D\_mid}-\xi_{\left(i+1\right)\_3D\_mid}\right)\cdot \left(\xi_{\left(i+3\right)\_3D\_mid}-\xi_{\left(i+2\right)\_3D\_mid}\right)}{{\left\|\xi_{\left(i+2\right)\_3D\_mid}-\xi_{\left(i+1\right)\_3D\_mid}\right\|}_{2}{\left\|\xi_{\left(i+3\right)\_3D\_mid}-\xi_{\left(i+2\right)\_3D\_mid}\right\|}_{2}} \end{array}$
The calculation steps for the coordinates of each point at $\left(b_{mid}+h\right)$ and the total curvature are the same as above. **Step 2:** Update the split interval, if $\Theta_{mid}>\Theta_{\left(mid+h\right)}$, then $b_{left}=\left(b_{mid}+h\right)$; otherwise $b_{right}=b_{mid}$. **Step 3:** If the interval of the intercept value is below the set threshold $\left(\left(b_{right}-b_{left}\right)<th\right)$, exit the loop. **Step 4:** The actual intercept of the two-dimensional projection of the target’s short-term straight flight trajectory is $\left(b_{left}+b_{right}\right)/2$; **Step 5:** Solve the three-dimensional coordinates of the target position: $\xi_{i}$, $\xi_{\left(i+1\right)}$, $\xi_{\left(i+2\right)}$, $\xi_{\left(i+3\right)}$.

**Table 3:** Simulation experiment’s theoretical value settings.

Parameter	Value	Unit
The azimuth range of the target relative to the receiver	$\left(10,\;80\right)$	deg
The elevation range of the target relative to the receiver	$\left(33,\;62\right)$	deg
The distance range of the target relative to the receiver	$\left(116,\;149\right)$	km

**Table 4:** Level division of bistatic distance errors.

	Low	Medium	High
Error magnitude/m	$\sigma_{\Delta R}\leq 100$	$100<\sigma_{\Delta R}\leq 200$	$\sigma_{\Delta R}>200$

**Table 5:** Level division of azimuth angle errors.

	Low	Medium	High
Error magnitude/deg	$\sigma_{\Delta \theta }\leq 0.5$	$0.5<\sigma_{\Delta \theta }\leq 2$	$\sigma_{\Delta \theta }>2$

**Table 6:** Mean ranging RMSE under different error combinations.

$\sigma_{\Delta R}$/m	$\sigma_{\Delta \theta }$/deg	Mean Ranging RMSE/m
100	0.5	326.47
100	2	553.09
100	4	818.95
200	0.5	522.85
200	2	694.01
200	4	1153.82
400	0.5	786.94
400	2	912.17
400	4	1397.85

## Data Availability

The data supporting this study are included within the article.

## Abbreviations

The following abbreviations are used in this manuscript:

TDOA	Time Difference of Arrival
FDOA	Frequency Difference of Arrival
DPD	Direct Position Determination
MIMO	Multiple Input–Multiple Output
SDR	Semidefinite Relaxation
DoA	Direction of Arrival
DoD	Direction of Departure
MUSIC	Multiple Signal Classification
GEO	Geostationary Earth Orbit
ECEF	Earth-Centered Earth-Fixed
UKF	Unscented Kalman Filter
PF	Particle Filter
RMSE	Root Mean Square Error
ANOVA	Analysis of Variance
